# Standard Units for Cannabis Dose: Why is it Important to Standardise Cannabis Dose for Drug Policy and How Can we Enhance its Place on the Public Health Agenda?

**DOI:** 10.1192/j.eurpsy.2022.46

**Published:** 2022-09-01

**Authors:** H. López-Pelayo, M. Balcells-Oliveró, A. Gual, S. Matrai, C. Oliveras

**Affiliations:** 1 Hospital Clínic de Barcelona, Consultation Liaison Psychiatry, Barcelona, Spain; 2 Hospital clinic, Addictions Unit, Barcelona, Spain

## Abstract

Regular cannabis use is associated with several adverse health outcomes including psychosis and cannabis use disorders. In the last decade, prevalence of last-month cannabis use increased by 27% and rate of treatment demand rose from 27 to 35.1 per 100000 inhabitants in Europe. Cannabis legal status is changing worldwide, and recently two European countries (Malta and Luxembourg) legalized its production, sales and use. Even United Nations withdrew cannabis from Schedule IV of the Single Convention on Narcotic Drugs (retained in Schedule I). This new scenario aligns cannabis more closely with alcohol, prescribed drugs or tobacco than illegal drugs. Implementing prevention and harm reduction strategies will be even more relevant in the nearly future. Based on the history with alcohol or tobacco, frequency of use alone misestimates the risks due to limited capture of variations of quantity per day of use on regular users. Standardisation of cannabis use has been proposed in the literature as an instrument to assessing the level of risk. In the Lisbon Addictions Conference (2019) a group of 32 professionals from 13 countries and 10 disciplines participated in a back-casting foresight exercise aiming to addresses challenges of implementation of SJU. They proposed three key steps in the implementation of SJU: 1) constitution of task-force for an evidence-based approach to SJU; 2) expanding the available data on cannabis-related risks; 3) examining the relationship between ‘risky use’ and SJU.

**Disclosure:**

No significant relationships.

